# HSP90 Inhibitors Modulate SARS-CoV-2 Spike Protein Subunit 1-Induced Human Pulmonary Microvascular Endothelial Activation and Barrier Dysfunction

**DOI:** 10.3389/fphys.2022.812199

**Published:** 2022-03-21

**Authors:** Ruben Manuel Luciano Colunga Biancatelli, Pavel A. Solopov, Betsy Gregory, Yara Khodour, John D. Catravas

**Affiliations:** ^1^Frank Reidy Research Center for Bioelectrics, Old Dominion University, Norfolk, VA, United States; ^2^School of Medical Diagnostic & Translational Sciences, College of Health Sciences, Old Dominion University, Norfolk, VA, United States

**Keywords:** SARS-CoV-2, spike protein, endothelial dysfunction, heat shock proteins, HSP90 inhibitors, AUY-922, AT13387

## Abstract

Severe acute respiratory syndrome coronavirus-2 (SARS-CoV-2) has caused more than 5 million deaths worldwide. Multiple reports indicate that the endothelium is involved during SARS-Cov-2-related disease (COVID-19). Indeed, COVID-19 patients display increased thrombophilia with arterial and venous embolism and lung microcapillary thrombotic disease as major determinants of deaths. The pathophysiology of endothelial dysfunction in COVID-19 is not completely understood. We have investigated the role of subunit 1 of the SARS-CoV-2 spike protein (S1SP) in eliciting endothelial barrier dysfunction, characterized dose and time relationships, and tested the hypothesis that heat shock protein 90 (HSP90) inhibitors would prevent and repair such injury. S1SP activated (phosphorylated) IKBα, STAT3, and AKT and reduced the expression of intercellular junctional proteins, occludin, and VE-cadherin. HSP90 inhibitors (AT13387 and AUY-922) prevented endothelial barrier dysfunction and hyperpermeability and reduced IKBα and AKT activation. These two inhibitors also blocked S1SP-mediated barrier dysfunction and loss of VE-cadherin. These data suggest that spike protein subunit 1 can elicit, by itself, direct injury to the endothelium and suggest a role of HSP90 inhibitors in preserving endothelial functionality.

## Introduction

The severe acute respiratory syndrome coronavirus-2 (SARS-CoV-2), responsible for the 2020 pandemic, has infected more than 250,000,000 people and provoked the deaths of more than 5 million people worldwide (John Hopkins Coronavirus Resource Center)^1^. SARS-CoV-2 enters the cell through its surface spike protein (SP) that binds the angiotensin-converting enzyme-2 (ACE2) expressed on alveolar type II and endothelial cells (Kumar et al., 2020; Zamorano Cuervo and Grandvaux, 2020). Multiple reports have underlined the contribution of endothelial dysfunction in SARS-CoV-2-related disease (COVID-19) (Lei et al., 2021). Indeed, COVID-19 patients display high rates of pulmonary embolism, thrombosis, and disseminated intravascular coagulopathy (Gando et al., 2016; Santulli, 2016, 2018), all mortality predictors in the critically ill, infected patients (Walborn et al., 2019).

The SARS-CoV-2 SP shares about 76 and 97% of amino acid identities with SARS-CoV and the bat coronavirus, RaTG13, with the receptor-binding domain (RBD) having about 74% and 90.1% homology, respectively. SP exists in trimers on the virus surface, with an average expression of ∼96 trimers per virus particle (Ke et al., 2020). SPs are typical class I viral fusion proteins that require protease cleavage for the activation of their fusion potential (Ou et al., 2016).

The ∼180 kDa SP contains two subunits, namely, S1 (∼75 kDa) and S2 (∼100 kDa), that mediate attachment and membrane fusion, respectively. The S1 *C-* and *N-*terminal domains fold as two independent domains, and even though the C-domain preferentially binds ACE2, both can serve as RBD (Li et al., 2005). Cleavage of the SP occurs in two steps: first, a cleavage between S1 and S2 followed by a second S2 cleavage. This process is mediated by different host proteases, such as furin, trypsin, cathepsin, transmembrane protease serine protease-2 (TMPRSS-2), TMPRSS-4, PIK5, TPC2, cathepsin L, or human airway trypsin-like protease (HAT) (Bertram et al., 2011, 2013; Gierer et al., 2013; Millet and Whittaker, 2014). Once SP is cleaved, the S2 binds ACE2 and promotes the fusion of the viral membrane to the host cell and the subsequent release of genetic material (Walls et al., 2020). Up to 45% of the cleaved subunit 1 (S1SP) is released as a peptide in the extracellular or vascular compartment (Ke et al., 2020). Little is known about the contribution of the S1SP in endothelial and organ injury in COVID-19 patients.

The S1SP is capable of eliciting endothelial inflammation and brain–blood barrier dysfunction (Buzhdygan et al., 2020), and we recently demonstrated that it can disturb human lung microvascular endothelial barrier function (Biancatelli et al., 2021).

Even though more than 750 clinical trials were initiated in 2020, only few interventions have shown promise (Recovery Collaborative Group et al., 2020). Thus, it is important to keep investigating the potential countermeasures that can block COVID-19-related endothelial dysfunction and ARDS.

Heat shock proteins (HSPs) are a family of chaperones that assist a large number of “client proteins” in folding, stabilization, and, if irreversibly damaged, degradation (Hoter et al., 2018). HSPs participate in the stress response and are actively involved in stabilizing pathways leading to inflammation (Craig, 1985). HSP90 is critically involved in the pathogenesis of acute lung injury and endothelial dysfunction, and its inhibition represents an intriguing therapeutical approach (Antonov et al., 2008; Barabutis, 2020).

Thus, we investigated the potential mechanisms responsible for SP-mediated activation and barrier dysfunction in human microvascular endothelial cells (HMVEC) and the potential beneficial effects of pre- or post-treatment with HSP90 inhibitors.

## Materials and Methods

### Reagents

Red protein G affinity beads, RIPA buffer, and protease inhibitor cocktail were purchased from Sigma-Aldrich Corporation (St. Louis, MO). The HSP90 inhibitors, AUY-922 and AT13387, were obtained from Selleck Chemicals (Houston, TX). The BCA Protein Assay Kit was purchased from Pierce Co. (Rockford, IL), and Western blot membranes were purchased from GE Healthcare (Chicago, IL). All antibodies used in Western blots and immunoprecipitation have published immunospecificity data available online. Rabbit anti-AKT, anti-phospho AKT, anti-IKBα, anti-phospho IKBα, anti-STAT3, anti-phospho STAT3, anti-VE-cadherin, anti-occludin, anti-cofilin, and anti-phospho cofilin were purchased from Cell Signaling Technology (Danvers, MA). Mouse monoclonal anti-β-actin and anti-p53 (P8999) were purchased from Sigma-Aldrich Corporation, and secondary IRDye 800CW goat anti-rabbit (926-32211) and IRDye 680RD goat anti-mouse (926-68070) were purchased from LI-COR Biosciences (Lincoln, NE). For SDS-PAGE, Protogel (30% acrylamide mix) and TEMED were purchased from National Diagnostics (Atlanta, GA), Tris-HCl buffer was purchased from Teknova (Hollister, CA), 10% SDS and ammonium persulfate were purchased from Thermo Fisher Scientific, and protein dual-color standards and tricine sample buffer were purchased from Bio-Rad Laboratories.

### Cell Culture

In-house harvested human lung microvascular endothelial cells (HLMVEC) were maintained in M199 media supplemented with 20% FBS and antibiotics/antimycotics as described earlier (Catravas et al., 2010).

### Endothelial Barrier Function

Human lung microvascular endothelial cells were grown on electrode arrays (8W10E +), and endothelial barrier integrity was estimated by the electric substrate impedance sensing (ECIS) method, using an ECIS model 1600R ζθ from Applied Biophysics. Experiments were conducted when a stable resistance was maintained above 800 Ω as we have previously published (Colunga Biancatelli et al., 2021). The single time frequency (SFT) mode was selected at 4,000 Hz and at an interval time of 600 s. Experiments were performed in triplicate and repeated at least 3 times. Resistance values were collected and normalized for each well’s value at *t* = 0. Data are presented as means (± SEM).

### Protein Isolation and Western Blots

Confluent HLMVEC cultured in 100 mm Petri dishes were exposed to S1SP and/or HSP90 inhibitors or vehicles. For time-course analyses, cells were incubated with 20 nM S1SP for 4, 6, or 12 h. For pretreatment studies, cells were incubated with HSP90 inhibitors (e.g., 2 μM AUY-922, AT13387, or vehicle) for 4 h followed by 20 nM S1SP for an additional 4 h. For post-treatment studies, cells were exposed to 20 nM S1SP for 4 h and then treated with HSP90 inhibitors (e.g., 2 μM AUY-922, AT13387, or vehicle) for 4 h. At the end of the experiment, dishes were placed on ice and washed 2 times with ice-cold PBS. PBS was removed and ice-cold lysis buffer was added (RIPA, protease inhibitor cocktail 1:100). Cells were then scraped, and the cell suspension was transferred to a microcentrifuge tube. Tubes were retained at 4°C for 15 min under continuous agitation and spun for 10 min at 14,000 rpm, the pellet was discarded, and the supernatant transferred into new tubes. Proteins were quantified by BCA protein assay, and tricine buffer with 2% 2-mercaptoethanol was added at 1:1 ratio. Proteins were denaturated by boiling at 100°C for 10 min, and protein lysates were subjected to SDS-PAGE electrophoresis in 12% sodium dodecyl sulfate Tris-HCl gels. Proteins were transferred onto nitrocellulose membranes and blocked for 1 h at room temperature with 5% non-fat dry milk in Tris-buffered saline containing 0.1% Tween 20. Membranes were incubated overnight at 4°C with specific antibodies, washed 4 times, and incubated for 1 h at room temperature with the appropriate peroxidase-conjugated secondary antibody. Bands were detected by digital fluorescence imaging (LI-COR Odyssey CLx). Densitometric evaluation of the bands was made using ImageJ software (National Institutes of Health, Bethesda, MD). β-actin was used to normalize loading differences.

### Immunocytochemistry

Round glass coverslips (Thermo Fisher Scientific) were placed in 12-well plates, soaked in 70% ethanol for 15 min, and dried under the hood. The cover glasses inside the plate’s wells were covered with 1 ml of 0.2% gelatin and incubated at 37°C for 30 min. Excess gelatin was aspirated, and then 500 μl of the cell suspension containing 6 × 10^5^ HLMVEC was placed on top. Coverslips with 75–90% confluent HLMVEC were fixated in 4% paraformaldehyde in PBS for 10 min, washed 3 times, then subjected to permeabilization with 0.1% Triton-X 100 in TBS for 10 min, and subsequently triple-washed in PBS. Cells were blocked with 5% BSA in 0.1% TWEEN overnight at 4°C. On the following day, coverslips were moved to a wet camera and incubated with VE-cadherin antibody (Abcam, 1:50 dilution) in blocking buffer at 4°C for 24 h. After washing 5 times in PBS, secondary incubation was carried out with Alexa Fluor 488 goat anti-rabbit antibody (Thermo Fisher Scientific, dilution 1:500) in the dark at RT for 1 h and then washed 5 times. F-actin was visualized with Texas Red-X phalloidin (Life Technologies, diluted 1:300). At the counterstaining stage, cells were incubated with 300 μM DAPI for 5 min in the dark. One drop of mounting media (ProLong Gold, Thermo Fisher Scientific) was added on pre-cleaned microscope slides (Superfrost Plus, Thermo Fisher Scientific) and placed over the top of the coverslips facing down, extra PBS was removed from the edges, and final slides were placed in the dark at RT for overnight drying. Confocal microscopy analysis was performed with an Olympics Fluoview FV10i, and pictures were analyzed with ImageJ software (NIH).

### Statistical Analysis

Statistical significance of differences among groups was determined by one- or two-way ANOVA followed by Tukey’s *post hoc* or Bonferroni’s test using GraphPad Prism Software (GraphPad Software, San Diego, CA, United States). Differences among groups were considered significant at *p* < 0.05.

## Results

### SARS-CoV-2 S1SP Provokes a Time- and Dose-Dependent Endothelial Barrier Dysfunction

**FIGURE 1:**
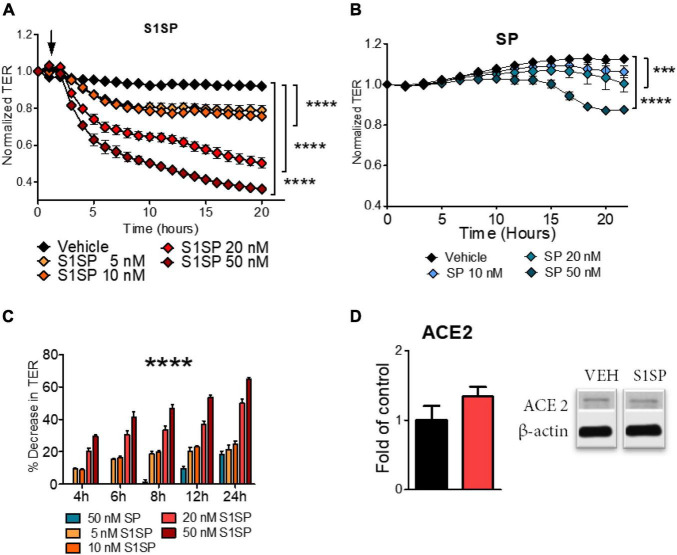
Barrier dysfunction of human lung microvascular endothelial cells (HLMVEC) exposed to SARS-CoV-2 intact spike protein (SP) or to subunit 1 (S1SP). **(A)** Changes in transendothelial electrical resistance (TER) of HLMVEC after exposure to vehicle or different concentrations of S1SP (5–50 nM). **(B)** Changes in TER of HLMVEC after exposure to vehicle or different concentrations of SP (10–50 nM). **(C)** Percentage decrease in TER at different time points after S1SP or SP. **(D)** Expression of ACE2 in lysates of HLMVEC exposed to S1SP for 4 h. Data represent means ± SEM; *n* = 3–5; ***p < 0.001; ****p < 0.0001 between groups, 2-way ANOVA and Bonferroni’s post-test **(A,B)**, or 1-way ANOVA and Tukey’s test **(C,D)**.

Human lung microvascular endothelial cells were grown on 810WE + arrays until a stable resistance was achieved (> 800 Ω). S1SP elicited a fast and concentration-dependent decrease in transendothelial electrical resistance (TER) (Figure 1A), while the intact SP elicited a delayed and much milder decrease (Figure 1B). As shown in Figure 1C, quantification of the percentage decrease in TER confirms time- and concentration-dependent relationships and demonstrates that the effect of 50 nM SP was equivalent to that of 5 nM S1SP, i.e., that HLMVEC were 10x more sensitive to S1SP than to SP. Analysis of ACE2 expression in HLMVEC displayed no significant changes after exposure to S1SP (Figure 1D).

### Subunit 1 of the SARS-CoV-2 Spike Protein Induces Endothelial Activation and Dysfunction

**FIGURE 2:**
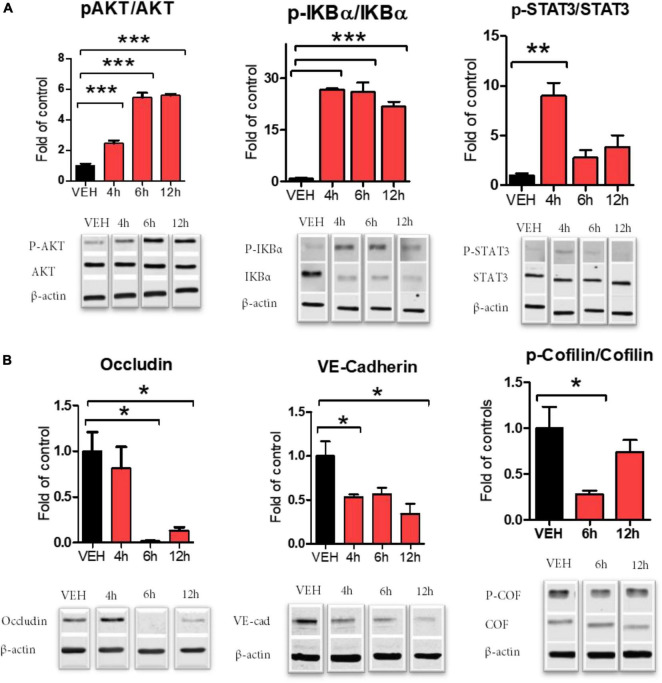
Activation of HLMVEC exposed to 20 nM of S1SP for 4–12 h. **(A)** Western blot analysis of AKT, IKBα, and-STAT3 phosphorylation; **(B)** cofilin phosphorylation and expression of occludin and VE-cadherin. Activated proteins are expressed as the ratio of phosphorylated to total protein. Data represent means ± SEM; *n* = 3–4; *p < 0.05; **p < 0.01; ***p < 0.001 between groups, 1-way ANOVA and Tukey’s *post hoc* test.

We investigated endothelial dysfunction after the exposure of cells to 20 nM S1SP. S1SP evoked robust inflammation mediated by early activation of STAT3 and NF-κB (Figure 2A). S1SP also increased time-dependently the activation (phosphorylation) of protein kinase B (AKT) (Figure 2A). We then analyzed changes in cytoskeletal rearrangements and the expression of junctional proteins. HLMVEC exposed to S1SP displayed dephosphorylation of cofilin and reduced expression of occludin and VE-cadherin (Figure 2B).

### HSP90 Inhibitors Prevent Subunit 1 of the SARS-CoV-2 Spike Protein-Induced Endothelial Barrier Dysfunction

**FIGURE 3:**
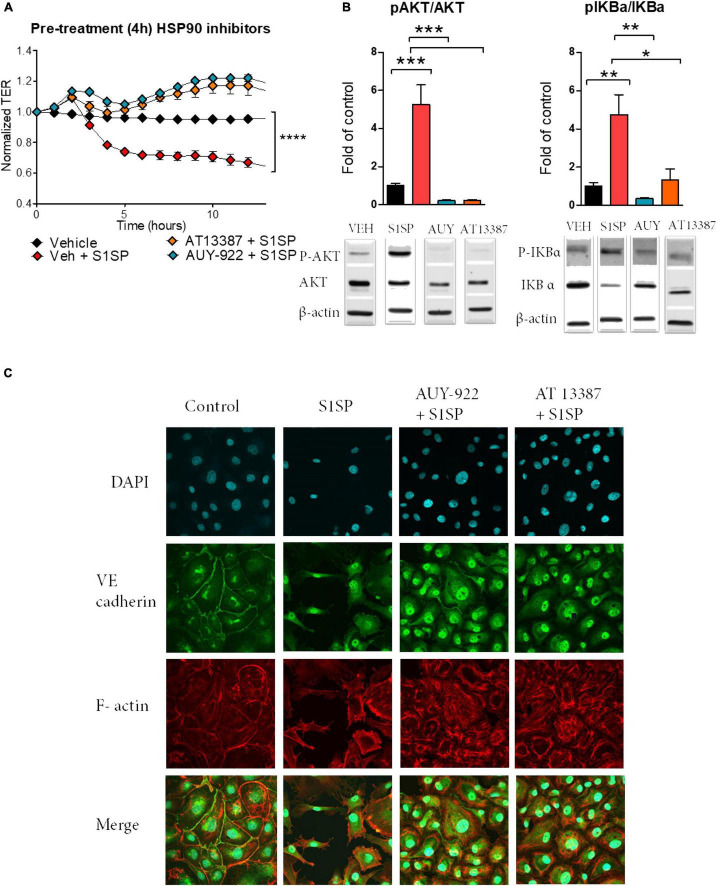
Pretreatment with HSP90 inhibitors prevented S1SP-mediated endothelial dysfunction. **(A)** 4 h of pretreatment with either HSP90 inhibitor (2 μM AUY-922 or AT13387), but not vehicle, prevented S1SP-mediated changes in transendothelial resistance. **(B)** Western blotting of activated (phosphorylated) and total AKT and IKBα from HLMVEC incubated for 4 h with HSP90 inhibitors before 4 h exposure to S1SP. **(C)** Immunostaining for VE-cadherin, F-actin, and DAPI of cells incubated 4 h with HSP90 inhibitors (2 μM) and subsequently exposed for 4 h to 20 nM S1SP. Means ± SEM; *n* = 3–4; *p < 0.05; **p < 0.01; ***p < 0.001 between groups, 1-way ANOVA and Tukey’s *post hoc* test.

We then investigated the ability of two chemically distinct HSP90 inhibitors, namely, AUY-922 and AT13387, to prevent S1SP-mediated injury. A 4-h pretreatment with either AUY-922 or AT13387 (2 μM) completely prevented S1SP-induced hyperpermeability (Figure 3A). Pretreatment with AUY-922 or AT13387 also prevented AKT and IKBα phosphorylation (activation) (Figure 3B) and loss of integrity of the monolayer, eliminated wide gap formation, and maintained the expression of VE-cadherin (Figure 3C).

### HSP90 Inhibitors Repair Subunit 1 of the SARS-CoV-2 Spike Protein-Induced Endothelial Activation and Barrier Dysfunction

**FIGURE 4:**
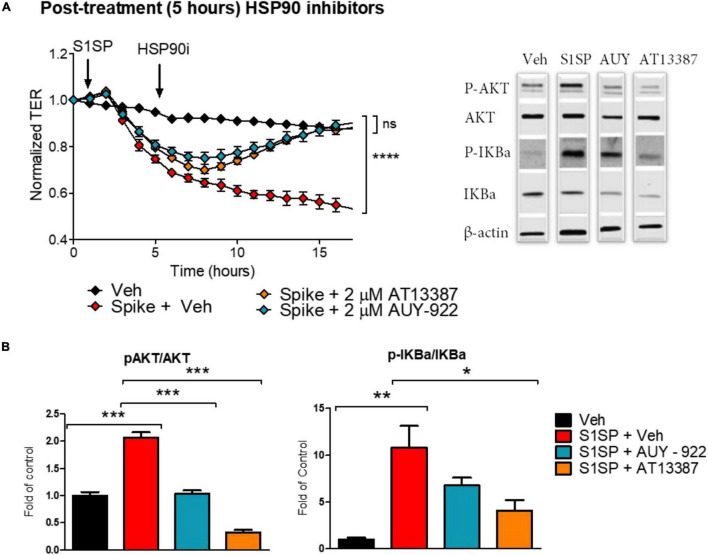
Posttreatment with HSP90 inhibitors restored S1SP-mediated endothelial activation and barrier dysfunction. **(A)** Post-treatment with either of the two HSP90 inhibitors (2 μM AUY-922 or AT13387) following 5 h of exposure to 20 nM S1SP repaired changes in TER. **(B)** Western blot analysis of activated (phosphorylated) and total AKT and IKBα in HLMVEC incubated for 5 h with S1SP followed by 4 h treatment with HSP90 inhibitors. Means ± SEM; *n* = 3–4; *p < 0.05; **p < 0.01; ***p < 0.001 between groups, 1-way or 2-way ANOVA and Tukey’s or Bonferroni’s *post hoc* test.

We also investigated whether post-treatment with HSP90 inhibitors could repair S1SP-induced endothelial barrier dysfunction and activation. HLMVEC seeded on gold electrodes were exposed to 20 nM S1SP for 5 h, at which time 2 μM AUY-922 or AT13387 or vehicle was added. Post-treatment with either AT13387 or AUY-922 completely restored endothelial barrier function after S1SP injury (Figure 4A). Furthermore, both AUY-922 and AT13387 repaired the S1SP-mediated activation of IKBα and AKT (Figure 4B).

## Discussion

Severe acute respiratory syndrome coronavirus-2 pathophysiology is not completely understood. COVID-19 patients develop atypical features of acute respiratory distress syndrome (ARDS) (Gattinoni et al., 2020a,b). These particular phenotypes exhibit reduced responsiveness to standard treatments, and high-flow nasal cannula and non-invasive ventilation are preferred over a more invasive management even in severe cases (Grieco et al., 2021; Shoukri, 2021).

The alveolar type II cells are not the sole targets of SARS-CoV-2-mediated injury. COVID-19 patients display increased coagulability, with autopsy findings of microthrombi in lung capillaries, which are considered the major determinants of death (McFadyen et al., 2020; Chen and Pan, 2021). Endothelial cells exposed to the spike protein display increased expression of adhesion molecules, and spike protein similarly promotes platelet activation leading to thrombophilia observed in COVID-19 (Buchrieser et al., 2020; Bussani et al., 2020; Buzhdygan et al., 2020; Passariello et al., 2021). Thus, the entire alveolar-capillary structure is involved during SARS-CoV-2 infection as reflected in ventilation/perfusion mismatch and in the various clinical features of near-normal and reduced lung compliance (Santamarina et al., 2020).

We investigated if subunit 1 of S1SP, released in the extracellular or vascular compartment after its interaction with ACE2, could participate in pulmonary endothelial inflammation and barrier dysfunction. In this study, the intact SP elicited a minor and delayed endothelial dysfunction response, in agreement with studies showing that endothelial cells are resistant to intact SARS-CoV-2 infection (Ahmetaj-Shala et al., 2020). This is not surprising, as the SP requires a two-step cleavage by furin and/or furin-like proteases. In this study, we focused on understanding if the S1SP, released after SP cleavage during cell infection, participates in eliciting a local inflammatory response.

Our findings suggest that S1SP exhibits high biological activity and is capable of damaging, in a time- and concentration-dependent manner, the integrity of HLMVEC. This was reflected in the activation of inflammatory pathways STAT3 and NF-κB; the phosphorylation of AKT and cofilin; and the loss of critical junctional proteins, occludin, and VE-cadherin.

The S1SP does not affect the constitutive expression of ACE2; similarly, various authors have provided evidence of a non-receptor-mediated pathway of S1SP-mediated injury. Spike protein can activate macrophages *via* Toll-like receptor 2 through NF-κB activation and the consequent release of IL-8, IL-6, and TNFα (Dosch et al., 2009). The non-receptor-mediated injury by S1SP is also demonstrated by its ability to permeabilize membranes and disrupt lipid bilayers (Asandei et al., 2020). Macrophages, exposed to the intact spike protein (SP), display increased levels of reactive oxygen species (ROS) and monocyte chemoattractant protein-1 (MCP-1) and, at high SP concentrations (100 nM), caspase activation and apoptosis (Barhoumi et al., 2021). Endothelial cells, transfected with a pseudovirus expressing the spike protein, displayed impaired endothelial function and NO synthase (eNOS) activity that reduced endothelium-dependent vasodilation in response to acetylcholine (Lei et al., 2021). This, in addition to the spike protein subunits 1- and 2-mediated overexpression of intracellular adhesion molecule-1 (ICAM-1) and vascular adhesion protein-1 (VCAM-1), suggests a direct role of the SP in eliciting a pro-inflammatory and pro-thrombotic vascular disease (Buzhdygan et al., 2020).

Accumulating evidence supports the notion that HSP90 inhibitors may serve as a novel, broad-targeted approach for the management of lung injury and ARDS. HSP90 stabilizes multiple proteins and thus optimizes or activates various crucial intracellular inflammatory pathways. When HSP90 is inhibited, its client proteins become unstable and degraded, with the result of inefficient or absent inflammatory signaling. Pretreatment or post-treatment with HSP90 inhibitors modulates the severity of lung injury and prevents mortality after exposure to LPS (Chatterjee et al., 2007). This is due, at least in part, to the fact that HSP90 interacts with NF-κB signaling at multiple levels: it regulates NF-κB by inducing kinase (NIK) (Qing et al., 2007) and together with HSP70 modulates IKK complex altering NF-κB activation (Salminen et al., 2008). Additionally, our group has demonstrated two mechanisms of HSP90/NF-κB interaction: the first mechanism is mediated by HSP90 binding to type-2 sirtuin histone deacetylase (Thangjam et al., 2016), and the second mechanism is mediated by HSP90 binding to the co-activator cAMP response element-binding protein required for RNA transcription (Thangjam et al., 2014).

In this study, the two HSP90 inhibitors, namely, AT13387 and AUY-922, effectively modulated the S1SP-mediated activation of IKBα, both when administered as pretreatment or as post-treatment.

HSP90 inhibitors display the ability to prevent endothelial activation at multiple levels. HSP90 inhibitors prevent the activation of p53 and RhoA and regulate the balance between phospho- and pan-cofilin, an effect mediated by LIM kinase 1 (LIMK1) (Li et al., 2006). The HSP90 inhibitor 17-DMAG modulates the phosphorylation of myosin light chain reducing LPS-mediated cytoskeletal rearrangements in bovine endothelial cells (Barabutis et al., 2019), and, more recently, we showed that AUY-922 was highly beneficial in an HCl model of endothelial dysfunction (Colunga Biancatelli et al., 2021). In this study, the use of two different third-generation HSP90 inhibitors ameliorated the loss of intercellular VE-cadherin reducing S1SP-mediated loss of integrity and hyperpermeability.

The novel approach with HSP90 inhibitors has proven to be beneficial in the treatment of different models of lung injury, including the spike protein subunit 1-mediated endothelial dysfunction. HSP90 inhibitors could represent a novel approach for ALI, and ARDS can reduce capillary permeability during inflammation and prevent leaks into the lung parenchyma and alveolar space. Additional data are required for further experimentation of HSP90 inhibitors in *in vivo* models of COVID-19.

## Data Availability Statement

The raw data supporting the conclusions of this article will be made available by the authors, without undue reservation.

## Author Contributions

RCB and JC conceptualized and validated the study. PS, BG, YK, RCB, and JC analyzed the study. PS and RCB investigated the study. JC contributed to resources, writing, reviewing, editing, and funding acquisition. RCB wrote original draft. All authors have read and agreed to the published version of the manuscript.

## Conflict of Interest

The authors declare that the research was conducted in the absence of any commercial or financial relationships that could be construed as a potential conflict of interest.

## Publisher’s Note

All claims expressed in this article are solely those of the authors and do not necessarily represent those of their affiliated organizations, or those of the publisher, the editors and the reviewers. Any product that may be evaluated in this article, or claim that may be made by its manufacturer, is not guaranteed or endorsed by the publisher.

## Footnotes

1
www.jhu.edu
